# Behavioral science labs: How to solve the multi-user problem

**DOI:** 10.3758/s13428-024-02467-4

**Published:** 2024-08-12

**Authors:** Diederick C. Niehorster, Marianne Gullberg, Marcus Nyström

**Affiliations:** 1 Lund University Humanities Lab, Lund University, Lund, Sweden; 2 Department of Psychology, Lund University, Lund, Sweden; 3 Centre for Languages and Literature, Lund University, Lund, Sweden

**Keywords:** Computer setup, Multi-user, Eye-tracking, Data safety, Remote management

## Abstract

When lab resources are shared among multiple research projects, issues such as experimental integrity, replicability, and data safety become important. Different research projects often need different software and settings that may well conflict with one another, and data collected for one project may not be safeguarded from exposure to researchers from other projects. In this paper we provide an infrastructure design and an open-source tool, labManager, that render multi-user lab facilities in the behavioral sciences accessible to research projects with widely varying needs. The solutions proposed ensure ease of management while simultaneously offering maximum flexibility by providing research projects with fully separated bare metal environments. This solution also ensures that collected data is kept separate, and compliant with relevant ethical standards and regulations such as General Data Protection Regulation (GDPR) legislation. Furthermore, we discuss preconditions for running shared lab facilities and provide practical advice.

## Introduction

Almost every lab facility suffers from an important usability and replicability problem: when any part of the hardware or software of an experimental setup is updated, previous experiments may fail to run, or worse yet, run but with changed behavior unbeknownst to the researcher. While this is already an issue for setups used by only a single researcher who may wish to run an old experiment, the issue is much worse for experimental setups shared by multiple users and for department- or university-wide lab facilities that cater to many simultaneous users. In these latter situations there is not only a problem of replicability. Even worse, different users’ potentially conflicting software or configuration needs may preclude the sharing of resources or engender error-prone data collection practices. Moreover, shared lab facilities must also solve the issue of storing collected experimental data in a General Data Protection Regulation (GDPR)-compliant and ethical way that guarantees data safety.

In this paper, we draw on extensive experience from managing the Lund University Humanities Lab, a university-wide shared lab facility for the behavioral sciences, in order to explore how behavioral science lab facilities might deal with the above and interconnected issues. The solutions presented in this paper are suitable for a minimal setup consisting of a single experiment station that caters to multiple concurrent research projects. However, to showcase the full potential of our solution for more complex facilities as well, in this paper we discuss the Lund University Humanities Lab’s Digital Classroom as an example case study. This facility involves additional complexity since it consists of multiple (16) experiment stations which for some use cases must work independently, but in others function as one large classroom-scale setup. We use the Digital Classroom facility as a case to provide advice regarding the practicalities of designing and running multi-user lab facilities, including a detailed description of the design and hardware infrastructure of this facility, the related staff and management policy, and procedural considerations that enable its operation. We furthermore present a newly developed management software tool that, together with the infrastructure of the digital classroom, enables us to provide separate environments for each research project using these facilities. This tool therefore resolves the problem of conflicting user needs and ensures data separation. We hope that this paper, the solution presented and the discussion, will help research units to set up and successfully operate multi-user lab facilities in the behavioral sciences.

### Practical problems when running multi-user lab facilities

Multi-user lab facilities face a series of important challenges in safeguarding experimental integrity, replicability, and data safety, but also usability for the users and manageability for the staff. In this section we will describe these issues in detail, and outline existing solutions. To facilitate the discussion, we will use scenarios from the Lund University Humanities Lab’s Digital Classroom (henceforth, the Digital Classroom).

**Fig. 1:**
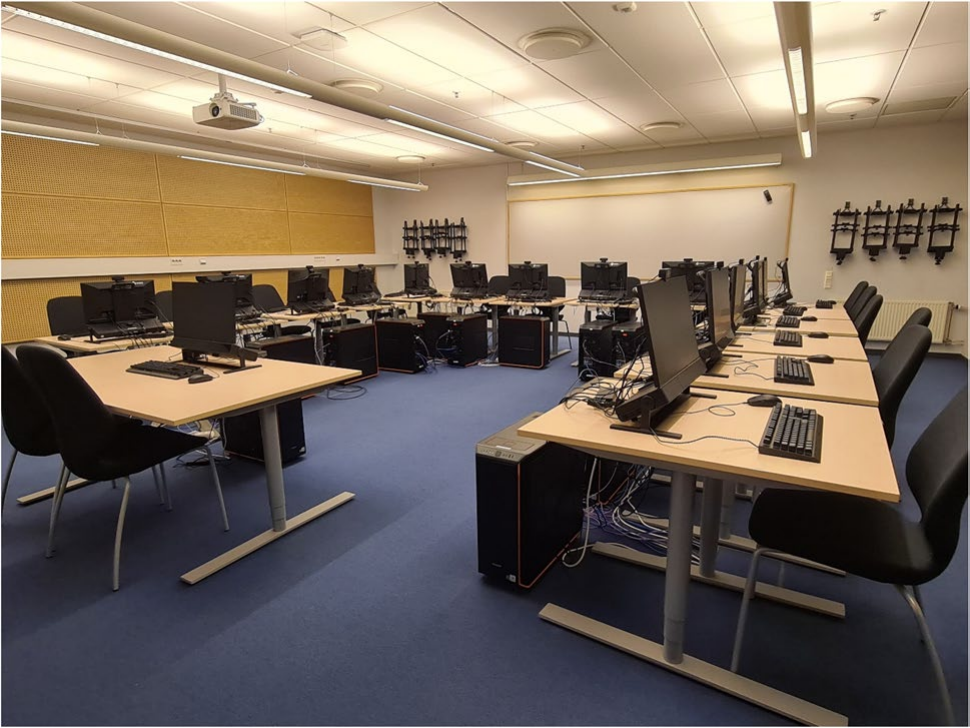
The Lund University Humanities Lab’s Digital Classroom, which consists of 16 eye-tracking stations connected over a wired network

The Lund University Humanities Lab is a university-wide and national-level shared lab facility that aims to provide state-of-the-art research equipment and expertise to facilitate research in the behavioral sciences. Since its inauguration in 2007, the Lab has developed routines that enable its primarily sensor-based facilities to be used simultaneously by many users with very different needs. One such multi-user facility is the Digital Classroom. The Digital Classroom (Fig. 1) is a full-scale classroom setup that consists of 16 computer stations equipped with eye-trackers, low-latency keyboards and mice, webcams, and microphones, alongside a computer infrastructure consisting, inter alia, of a master computer and a server that allow the room to be used as one integrated facility rather than a series of unconnected experimental stations. Such a facility offers the opportunity to conduct innovative interdisciplinary research such as studying the behavior of whole groups of people at once in, for instance, educational science (Oliva et al., 2016; Špakov et al., 2019) and cognitive psychology (Strukelj et al., 2016a; Oliva et al., 2017; Niehorster et al., 2019). Furthermore, many other research projects use the Digital Classroom solely as a convenient means to collect data more quickly since it allows for measurements to be made on multiple participants in parallel (e.g., Pärnamets et al., 2016; Strukelj et al., 2016b, Strukelj & Niehorster, 2018; Gidlöf et al., 2021; Dutceac Segesten et al., 2022; Johansson et al., 2022; Timoshenko-Nilsson, et al., 2023). Lastly, the Digital Classroom underpins the ability of the Humanities Lab to offer Master- and PhD-level eye-tracking courses as well as commissioned education for international researchers^1^ that includes a hands-on component.

### User scenarios

These different simultaneous uses of the Digital Classroom lead to a considerable challenge: how do we facilitate the realization of each of these research projects and educational uses with sufficient flexibility without creating a management and maintenance headache for the Humanities Lab staff? To facilitate this discussion, we first introduce five potential users of the Digital Classroom and their needs, and then examine what solutions can feasibly accommodate all their needs at the same time.

1. A computer science PhD student who is interested in studying collaboration patterns and tool use in software-mediated pair programming. The development environment and additional tooling that this student uses to conduct their study runs on Linux.
2. A visual arts researcher who is interested in the perception and emotional reception of Van Gogh paintings. They are new to using quantitative, sensor-based data acquisition methods and use off-the-shelf software on Windows to collect their data. Their data consists of both eye-tracking data and webcam recordings of the participants’ faces. The researcher has hired a research assistant (RA) to assist with part of the data collection. After a lengthy analysis and writing process, additional data collection is required years later to satisfy reviewers of the journal submission. A new RA helps with this data collection.
3. A social scientist who is interested in studying task division when performing visual search experiments in which pairs of participants can see each other’s gaze position. The researcher is interested in studying how the emergence of successful divisions of labor depends on the personality of the paired participants and therefore also collects data from a battery of personality, pro-sociality, and apathy questionnaires.
4. A communication scientist who is interested in political communication on social media platforms during the Swedish election season. Instead of a controlled setup, they wish to study what their participants are exposed to, and therefore perform screen recordings while participants scroll through their Facebook feed. They simultaneously employ eye-tracking to examine which of the political communications their participants attended to.
5. Two Humanities Lab employees who teach a hands-on eye-tracking course. This course uses a specially prepared Windows installation that contains all the software and scripts that the students will need during the course.

## User requirements on behavioral science lab facilities

In order for a behavioral science lab to be usable for each of the above users, it must fulfill a series of minimum requirements commensurate with their individual needs and activities. It must also offer an environment with good usability. Some of the requirements are common to most or all users, whereas some projects come with specific requirements or wishes unique to them. In this section we briefly list common requirements to which a lab environment for the behavioral sciences should conform. We return to these requirements later in the paper when evaluating the suitability of different potential solutions to the multi-user problem.

### Bare metal access to hardware (to meet timing requirements)

Users require “bare metal” access to local computer equipment, that is, data collection and experiment software should be able to directly access the hardware in the computer without intervening hypervisors, virtualization layers, or other types of indirection or remote execution. In order to achieve reliable results, behavioral science studies often have stringent timing requirements (Plant, 2016; Plant & Quinlan, 2013). To meet these requirements, extensive software solutions (Brainard, 1997; Pelli, 1997; Kleiner et al., 2007; Hwang et al., 2019; Hill et al, 2019; Peirce, 2007; see Strasburger, 2020), sometimes in combination with custom hardware solutions (e.g., Thurgood et al., 2011; Watson et al 2019), are used that need direct access to the hardware in the computer to function correctly.

Most of our potential users have this requirement. For instance, all users (1–4) except the teachers need accurate synchronization of the measurement equipment to the visual stimuli on the computer screen to be able to infer, e.g., the latency of participants’ reactions. Furthermore, the visual arts researcher (user 2) requires synchronized acquisition of data from an eye-tracker and a web camera, and the cognitive and computer scientists (users 1 and 3) require near real-time communication between multiple experimental stations with known latency.

### No restrictions on software choice

Users should have minimal restrictions on which software they can use for building their experiments. There are many different commercial and open-source solutions available for building behavioral science experiments. The choice between software packages can vary widely between projects, depending, for instance, on which features are required for the project, the researchers’ programming ability, their previous experience with software packages and any previous work they wish to build on, and existing preferences among researchers in their scientific field. Restrictions on what software can be used in a lab creates accessibility barriers when the requirements for some projects cannot be met, and can lead to increased risk of errors when researchers are forced to reimplement their experiments using the tools provided in a lab. In contrast, if users are able to freely install the software they need, this simplifies management of the facility and increases its usability.

This is a factor of importance for all of our users. Our users have different operating system requirements (the computer scientist [user 1] needs Linux, whereas the other users are likely to use Windows). Moreover, they also require different experiment software. For instance, whereas the visual arts researcher (user 2) will probably opt for the software that comes with the eye-trackers in the lab or a similar integrated graphical experiment environment, the cognitive scientist (user 3) will need to program their own experiment. The teachers (user 5) would greatly benefit from the ability to provide their students with a specially prepared environment containing everything the students need for their course.

### Adequate data security

Solutions must be in place for safeguarding data. In the past decade, requirements and regulations on the handling of data have been developed for academia, industry, etc. The ability to meet (inter-)national ethical standards and regulations such as the GDPR or the Health Insurance Portability and Accountability Act (HIPAA) in shared lab facilities will usually minimally require that the data from each project is stored in a secure and backed-up location that can only be accessed by project members. Leaving data on shared lab computers where anybody with access to the facility can reach it is a breach of multiple regulations.

Again, this is a factor of importance for most of our users. It should especially be noted that the cognitive and communication scientists (users 3 and 4) collect sensitive personal data (as defined by the GDPR), while the face videos collected by the visual arts researcher (user 2) will also require special handling to meet ethical standards.

### Replicability

For replicability of research results, it is paramount that the exact conditions in which a study was conducted and data acquired can be recreated. Users should therefore be offered the capability to “freeze” and store the exact software, configuration, and experiment setup of a given experiment station, and then be able to restore it at any later date. This capability is also vital in two further scenarios. First, to enable effective use of capital-intensive resources, lab facilities should be able to support multiple simultaneous data collections. The ability to store and deploy the exact experiment environment for each of these data collections is critical to allow multiple data collections to occur simultaneously without them interfering with each other. Second, replicability is crucial for data collections that involve multiple experiment stations. In this scenario, to ensure that each station is identically configured, users should be able to preserve and store the exact setup of a given station and deploy it to other stations.

All our potential users would do well to freeze and store their setup to be able to recreate it later. Nonetheless, several of the use cases specifically speak to the need for this capability. The visual arts researcher (user 2), for instance, needs to recreate their exact setup to collect additional data to satisfy their reviewers. Furthermore, the cognitive scientist (user 3) needs to set up multiple identical experiment stations for their collaboration study, and the teachers (user 5) need to be able to continue their teaching activity over several months, interleaving their use of the lab with that of other ongoing activities.

## Preconditions and considerations for multi-user lab facility operation

Besides the above requirements put on the lab facility by its users, the design of a lab facility requires planning to render management and facility maintenance practicable, and the facility attractive to users. Some factors to consider are similar to those discussed above, since they matter from both the user and the staff perspective.

### Hardware

For lab facilities that consist of more than a single experiment station, like our example classroom setup, an important precondition for practical management is to ensure as little variation in hardware between stations as possible. Dealing with identical hardware means that only one station needs to be manually set up or updated, after which all other stations can then be supplied with identical copies (e.g., using the store and restore functionality discussed under Replicability). This would not be possible if stations had different hardware, for instance due to different driver needs and software configuration. Furthermore, software may behave differently on different platforms (Yan et al., 2018). Having multiple identical stations additionally enables flexibility in resource allocation and decreases downtime for users should a single station fail. To illustrate this, consider a lab center with multiple identical experiment rooms, where any user can be assigned any of the rooms because identical hardware enables a portable software configuration.

### Software

For lab facilities to be able to support top-quality research, users should ideally have as few constraints as possible on use of the equipment, including the software they use. For such an option of maximum flexibility to be feasible and practicable, users should be provided with a basic installation that contains what most users need. More advanced users should also be able to manage themselves what software (and even operating system) they install on the experiment stations without relying on lab staff or an IT service unit. Making (more advanced) users self-sufficient in this manner avoids unnecessary administrative overhead for lab staff and, as indicated above, increases the usability of the facility.

### Support infrastructure

Above, we have argued that each user of the lab facility should have their own fully customizable environment that they can access from the experiment stations. The time it takes users to place their environment on the experiment stations should be short (say, 5–10 minutes maximally) to make such a system usable in practice and provide a good user experience. Achieving this requires either that all user environments are stored locally on each experiment station, or that there is a support infrastructure in place that enables fast delivery of the user environments to the experiment stations. A solution that stores all environments locally on all stations is unwieldy, and does not allow for flexible resource allocation where users are reassigned to other stations or where they can replicate their setup to other stations at will. Moreover, it increases the risk that experiment data and other confidential information such as program code can be accessed by others. In contrast, a solution without these drawbacks is one where user environments are stored elsewhere, such as on a central server, and are then delivered to experiment stations on demand after user authentication. However, it does require a fast local network and a server infrastructure capable of delivering the user environments over the network at sufficient speed. This central storage infrastructure must furthermore be secure and enable easy access for users to their setups and their collected data.

### IT security

There are several aspects of IT security to be considered by facility management. We discuss these below.

#### Access management

To maintain security of both experiment code and collected data, and to ensure that only authorized users can access the facility, an access management system is needed. This system needs to be flexible, allowing for multi-user projects. Organizing such a system by project is recommended so that all staff involved in a project can access its experiment setup and collected data. For instance, the visual arts researcher and the teachers (users 2 and 5) have activities that consist of multiple users (RAs for the researcher and all teaching staff for the teachers) who need access to their project’s setup. Ideally, the projects and the authorized users associated with them are registered in the host institute’s credential system such that users do not need separate user accounts and passwords to access the lab facility and their projects. This solution increases usability from the user’s perspective and decreases administrative overhead for lab staff.

#### Virus scanners and firewalls

Computer equipment often has software that protects against malware such as viruses (virus scanners) as well as software that protects against unauthorized intrusions over the network (firewalls). However, such software is incompatible with the need of behavioral science experiments for unrestricted direct access to hardware and deterministic timing, since they often form additional layers between user software and the computer’s operating system that may hold up software processes for indeterminate amounts of time. Therefore, it is common practice to deactivate such software on behavioral science experiment machines. However, this means that special care must be taken in the design of infrastructure and user policies to prevent malware from entering and spreading in the lab. Such policies could, for instance, include rules against the use of removable media such as USB sticks which may be infected with viruses and restrictions on internet access (see Internet access).

#### Data safety and privacy regulations

The lab facility should be set up such that users can meet relevant data safety and privacy regulations, as well as ethics requirements. This will minimally entail providing users with a secure and backed-up storage location for their collected data that resides elsewhere than on the experiment station computers in the lab. Furthermore, it entails a system that secures access to experiment stations such that only project members can access the contents of an experiment environment that may have inadvertently been left on a lab experiment station. Together, these two measures should minimize the risk for data leaks, and allow users to appropriately secure access to their data.

#### Internet access

For some experiments, users need access to the internet. In our example, the communications scientist (user 4) needs internet access, and the students attending the course of the teachers (user 5) would probably also benefit from internet access to be able to retrieve relevant literature and access their email and the course platform. However, opening a lab infrastructure to the internet brings data safety and experiment integrity risks. Firstly, when the internet is accessible from experiment machines, this opens an additional route through which viruses can enter the lab infrastructure, and potentially rapidly spread. It is not hard to imagine a scenario in which ransomware encrypts or exfiltrates the data from multiple projects. As discussed, this risk is compounded by the common practice of disabling resource-intensive gatekeeper software such as virus scanners and firewalls on experiment machines to meet the stringent timing requirements of behavioral science experiments.

A second reason that internet access may not be desirable on experiment machines is that automatic updates of the operating system or other experiment software may compromise the integrity of the experiment. Behavioral scientists are expected to verify correct operation of their experiment where relevant, such as the content and timing of their stimuli and response acquisition equipment, for instance by carefully calibrating their stimulus delivery equipment such as monitors and headphones. Yet updates of software may affect the functionality of any of these aspects of the experiment, introduce new bugs, or solve bugs that the experiment has worked around, leading to issues such as changed stimuli or incorrect timing, possibly without the experimenter being aware of it. It is therefore good practice *not* to perform updates of the operating system or the experiment software during the data recording period of a project. But when experiment machines are connected to the internet, such updates may happen automatically.

In sum, it is important when planning a lab facility to carefully consider whether experiment machines should be connected to the internet by default, and whether it should be possible to connect them at all. In exceptional circumstances where internet access is required for a project, it may be granted, but it will require additional safety steps to be taken to isolate any connected machines.

### User policy

For shared lab facilities to thrive, it is important that all their users understand the rules of engagement. Lab management should therefore consider developing a code of conduct for users and have induction meetings with users where it is explained before granting access. It is useful to formalize this by means of a user agreement that specifies the responsibilities of the lab facility to the user, and the user’s responsibilities toward the lab facility. A code of conduct and user agreement could include items such as the facility booking system and booking etiquette (e.g., only book timeslots that you will actually use), clarifications of (limits to) lab staff responsibilities (e.g., ensure correct functioning of the equipment, but not responsibility for users having an appropriate experimental design), usage instructions and etiquette for the lab facilities, data storage responsibilities for each party, and proper acknowledgement of the lab facility in resulting publications.

### Staffing

Running a lab facility requires more than equipment and staff who make sure equipment works. It is vital to carefully consider staff expertise when recruiting. To create an attractive environment to users, it is important that staff are also experts in the provided lab equipment and related experimental methodology (i.e., have research experience of their own), and that staff with the competencies required for administering the general lab computer and network infrastructure are available. It may furthermore be desirable that lab staff offer training in the lab equipment and associated research methods. This serves the double purpose of increasing accessibility to the lab facility, and ensuring that users have a minimum level of competence. This optimizes the gain from the considerable resources invested in the lab facility. Ideally, staff positions should include a percentage of research time and support for attending relevant conferences in order to ensure that staff remains well informed about the state of the art. Lastly, to ensure sustainability and reduce vulnerability of the lab, it is important that key knowledge about lab setup, infrastructure, and tools developed in the lab is both well documented and distributed across several staff members. Failure on this front leads to vulnerability and could lead to loss of expertise and to the need to close down facilities in cases of illness, change of staff positions, etc.

## Available solutions

The sharing of computational and experimental hardware resources is of course not a new notion, and various approaches and solutions exist. Next, we will evaluate existing solutions for their suitability to the scenario we have sketched. We discuss these solutions grouped by category. While parts of this discussion may appear to be unnecessarily in-depth, this is because one of the aims of this section is to arm the non-technical manager against unsuitable solutions that an IT department may suggest. As discussed above, behavioral science studies impose stringent requirements on experiment stations and the software that runs on them, and these are often in direct opposition to the drive of IT departments to standardize and remotely manage computer systems.

### The “don’t worry about it” approach

A common “solution” to the above problems is to simply ignore them. Users of experimental equipment are left to fend for themselves, while some responsible staff member occasionally cleans up and updates the software on the machine. Users manage their data through some folder structure on the hard drive of the experiment machine that can be accessed by anyone with access to the machine. As argued above, this approach offers no data security and it runs afoul of ethical standards and privacy regulations. Since users easily get in each other’s way, this mode of operation furthermore threatens experiment integrity. For example, two users that need different settings on the data acquisition hardware, the operating system, or some software on the operating system each need to go through an elaborate checklist before starting a data collection session to ensure that everything is set up as needed for their experiment. Mistakes are easily made in this process, compromising the collected data. In worse scenarios, users are not aware that another user has changed a setting that compromises their experiment (we have seen this happen), and collect data that unbeknownst to them is faulty. Obviously, data collected on equipment managed in this manner are often not replicable.

### The “lock down everything” approach

A solution that prevents users from interfering with each other is to lock down everything on the experiment machines, to the extent where users cannot install or upgrade any software themselves or change any settings. This approach may be familiar to users of computers that are managed by a central IT unit or a bossy lab manager. While users of such facilities are somewhat safer regarding inadvertent changes of settings or software that compromise their experiment, assuming that any changes made are communicated, they have no flexibility to set up the system to meet their requirements. That means either that bespoke research cannot be performed in the facility because the straitjacket of prescribed tools is too constraining, or that users spend significant time working with a staff member to set up the system to their specifications. This approach thus reduces the accessibility of the facility and causes significant overhead both for the staff and for the user. It also hinders iterative workflows where users test and adjust the setup until it meets their needs. It should also be noted that this approach does not necessarily address the issue of data security and that nothing is in place to ensure replicability of data for anything more than a short period of time.

### The cloud

Many scientific fields require significant computational and storage resources that do not have to be accessed in real time. For instance, in the biomedical sciences, there are initiatives such as SciLifeLab (www.scilifelab.se) and the Galaxy project (galaxyproject.org) that provide platforms where (distributed) research teams can store their data and perform their analyses. These platforms enable working in such a way that the analysis pipeline is fully traceable, providing certainty regarding which environment and data, research software, and processing steps taken in which order led to the reported results. For instance, PEGR (Shao et al., 2022) is a tool that integrates with the Galaxy platform for tracking genomics data acquisition and analysis in an open and fully documented way which supports reproducible science. Further examples of such platforms and infrastructures are APRICOT (Giménez-Alventosa, 2020) for the biomedical sciences, and the “Reproducible Experiment Platform” (Likhomanenko et al., 2015) for particle physics.

Computational resources are furthermore provided by many research institutes through on-premise hosting, or are accessible through commercial products such as Google Colab (https://colab.google) and Amazon SageMaker Studio Lab (studiolab.sagemaker.aws). While the above tools and platforms have made great strides towards enabling reproducible, collaborative, and efficient science in a number of fields, they are of little use for the typical behavioral science cases sketched in this paper since they focus on data processing, not acquisition, and offer no or very limited ability to interact with local hardware, to present graphics, sound, or other stimuli.

A thin client architecture, for instance, the solutions provided by Citrix Cloud (www.citrix.com/cloud.html) in hybrid mode, VMware Horizon (www.vmware.com/products/horizon.html), or Microsoft Remote Desktop Services (learn.microsoft.com/en-us/windows-server/remote/remote-desktop-services/welcome-to-rds), can alleviate part of these problems. In such a setup, the user’s desktop is hosted on central server(s), and most computation and generation of graphics is performed on these servers. The local thin client on the other hand is a low-performance system that acts as a terminal: it is responsible for establishing a connection to the server, showing the desktop or other graphics rendered on the server, and forwarding the input from user peripherals such as keyboards and mice back to the server. With such a design, each user’s experiment environment is isolated and can easily be accessed from any location, e.g., an experiment station, as required. Also, such solutions are capable of delivering stimuli such as graphics and sound. However, they are not designed for the kinds of operations required for behavioral science laboratories. Access to specialized local hardware attached to the client may be impossible, and any form of reliable timing of stimuli output or response acquisition is impossible due to the virtualization and network communication layers involved in these architectures.

Lastly, there are available cloud solutions that can be used for or are specifically geared toward data collection. Examples are Mechanical Turk (www.mturk.com), Pavlovia (pavlovia.org), and Gorilla (gorilla.sc). Such services are aimed specifically at rapid remote data collection and were for instance used with great success during the COVID-19 pandemic. They may seem like a great replacement for dedicated lab facilities, and may also be seen as making it easier to run different experiments in a computer lab. We briefly tackle both these points. First, when it comes to replacing lab facilities, it is important to realize that such services bring data collection into the home of participants, and run experiments on their own computers, wherever they are placed. Although this may be fine for some research, data collection that requires control over the environment of the participant, needs heavy computational resources, or requires specialized hardware cannot be executed using such platforms. Second, running experiments hosted on such services in a local computer lab is not a solution to the problems sketched above. Such a setup only adds an additional layer of complexity by hosting experiments online and running them in the browser, does not offer access to specialized local hardware, and does not mitigate any of the issues around changing software and settings discussed above.

### Virtualization

Another possible solution is to use virtualization, for instance of specific applications or of whole desktops. While the thin client solutions discussed above rely on virtual desktop environments that run remotely on a server, virtualized desktops can also be run directly on the experiment machines. Using desktop virtualization and appropriate network infrastructure would allow users to load and execute a virtual disk image with their experiment environment on any local machine with a suitable hypervisor and hardware installed. However, virtualization technology such as hypervisors add a layer of indirection between the data acquisition software and the hardware of the experiment station. Depending on the capabilities of both the hypervisor and the hardware (expensive workstation instead of consumer versions are often required), it is often entirely impossible to directly access the local hardware, or at minimum accurate synchronization of data acquisition to the presented stimuli is probably compromised. Popular experiment frameworks that focus on accurate timing, such as Psychtoolbox, will refuse to work inside a virtual machine.

Instead of virtualizing the whole operating system and desktop, only specific user applications can be virtualized, such as the software used for creating experiments and performing data acquisition. This capability is offered by products such as Microsoft App-V (learn.microsoft.com/en-us/windows/application-management/app-v/appv-for-windows) and VMware ThinApp (www.vmware.com/products/thinapp.html). Such solutions often involve packaging the application together with all it requires to run and all its settings in a container, which can then be streamed as needed to a client machine and run in a virtual machine. This gives users easy access to the applications they need, if they have been packaged once by lab staff. However, since applications have to be packaged by lab staff, this solution does entail administrative overhead for staff and users. It also does not allow for the creation of a persistent complete experiment environment for a user, since only individual applications are packaged in isolation from each other. Furthermore, operating system and driver settings and versions are not preserved for the user with this setup, which means that users may interfere with each other’s experiments and that setups are not replicable. Lastly, since these applications run in a virtual environment, the same caveats as for whole-desktop virtualization described above apply.

A third type of virtualization is operating system-level virtualization, which is also known as containerization. A popular product in this space is Docker. Such solutions provide separate containers that can contain one or multiple software programs and are allocated a part of the host machine’s resources. In contrast to the two types of virtualization discussed above, this compartmentalization is achieved without introducing a hypervisor or other level of indirection to access computational and other system resources. It has been successfully used for wireless networking experiments (Brunisholz et al., 2016). However, a problem for behavioral science use cases is that exclusive access to hardware on the host machine is often not possible (for instance, exclusively assigning the graphics card to a Docker container will lead to a black screen, as the host no longer has graphics resources), necessitating indirect access to most of an experiment station’s hardware. As with the other types of virtualization, this is not usable for experiment setups since timing and synchronization requirements cannot be met. Furthermore, in this paradigm the containers share the host operating system’s kernel, restricting choice of operating system (when using a Windows host, it is not possible to run a different version of Windows or a Linux system without introducing a virtual machine as an intermediate host), device configuration, and the storage of device or host operating system settings. Finally, to use this solution, users need to be comfortable with using a command line and extensive configuration files to manage their experiment container, or else need extensive staff support to be able to set up their data acquisition. This may be an unacceptable accessibility hurdle.

## Implementation: The Humanities Lab Digital Classroom

We now turn to the description of an implementation of a multi-user behavioral science environment that meets the discussed constraints. This is the Lund University Humanities Lab’s Digital Classroom (hereafter digital classroom), previously introduced above. The digital classroom, being a university-wide research infrastructure at Lund University, is not designed for the needs of a specific set of research projects. As service providers to a very wide user base, we cannot tailor the room to specific requirements, but instead must achieve maximum flexibility in what the setup can accommodate.

To provide this flexibility and meet all the goals described in the user requirements and preconditions sections above, we have developed a solution that allows each project to have completely separate environments. This is achieved by a system where users, upon entering the room, log in to a specific project. They are then provided with access to disk images that they can rapidly deploy to one or multiple stations in the room. To help users get started with setting up a project environment, they are provided with a base disk image of a clean Windows environment with common experiment software already configured. Users are then free to modify this Windows installation in any way they want or not use it at all. Users can store a disk image of their project environment at any time, for instance when they have completed it and want to distribute it to the other stations in the room, or simply store it for later use and reproducibility purposes, but also at key checkpoints during project setup.

Importantly, to make this system using disk images practically workable, we have set minimum standards for acceptable operation: creating an image of a station needs to be fast (we consider a time under 10 minutes to be acceptable in most situations), and deploying a disk image to a station is ideally even faster (since it occurs more frequently). Furthermore, to facilitate recordings involving multiple stations, we have carefully designed the system to enable close synchronization of the clocks of the experiment stations, and to provide for low-latency inter-station communication.

In this section we first describe the physical setup of the room and the surrounding hardware infrastructure. This is an important part of the setup, as the station, server and network hardware, and design enable meeting the performance and security requirements detailed above. We then discuss a new open-source software tool, labManager (https://github.com/dcnieho/labManager), that we have developed for managing multi-tenant behavioral research setups, and which is a critical part of making the digital classroom environment easy to manage while maximizing flexibility for its users and safeguarding reproducibility. Finally, we describe policies we have developed surrounding the use of the facility. Following this, we report on several tests to examine whether our design goals are met. Specifically, these tests examine what performance we achieve when creating a disk image and when deploying a disk image to all stations at once. We furthermore examine the communication latency between different experiment stations.

### The room

A picture of the room is shown in Fig. 1. Although some of the hardware and accessories in the room are specific to our setup and not required for setting up a similar facility, we will here provide a complete description that allows the reproduction of our design

#### Furniture

**Fig. 2:**
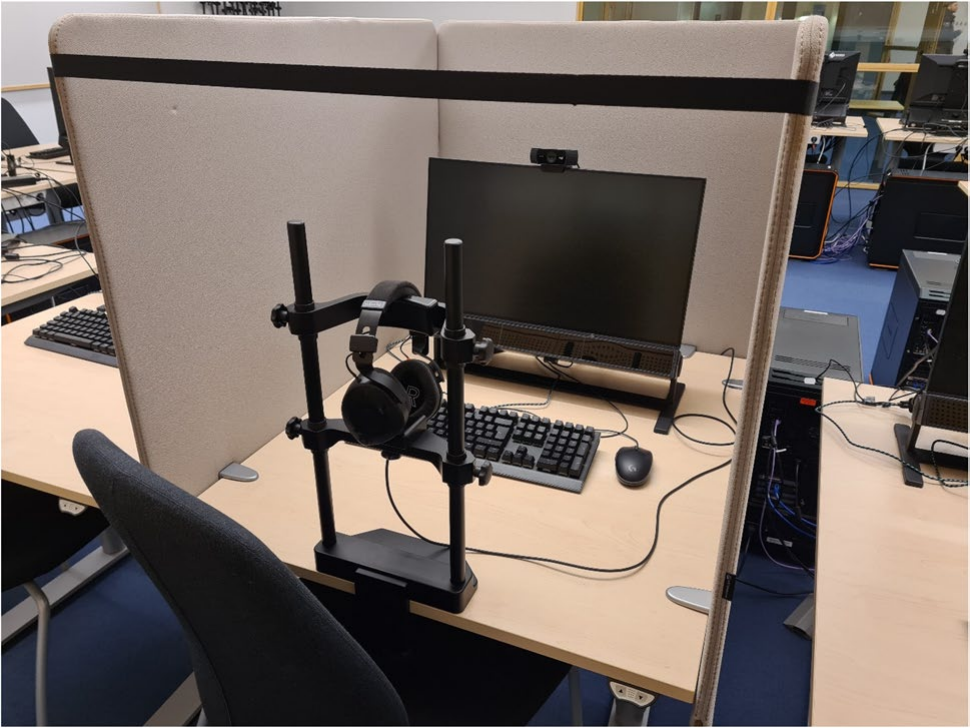
A single station in the digital classroom

 The digital classroom contains 17 computer stations, 15 identical student stations, one teacher station, and one further master station. The student stations are all designed around an 80 cm wide × 100 cm deep table with motorized height adjustment. This form factor is unusual, but the deeper tables provide the required flexibility for a setup involving remote eye-trackers that need to operate at a specific range of viewing distances. The only difference between the teacher and student stations is that the table of the teacher station is bigger, at 180 cm wide × 100 cm deep. To enable each station to be turned into its own booth and to remove visual distractions from other people in the room, customized 80 cm-high screens (Fig. 2) that can be attached to the sides and the back of each table are also available. Lastly, each station is provided with a chair with fixed legs that is not height-adjustable. We prefer a fixed chair in combination with a height adjustable table to minimize participants’ ability to fidget.

#### Computer stations

 The teacher and student stations are served by a computer built into a Be Quiet! Dark Base 900 computer case. This computer case, as well as the processor cooler (Be Quiet! Dark Rock 4), power supply (Be Quiet! Dark Power Pro P11), and graphics card (Asus GeForce RTX 2080Ti ROG Strix Gaming OC) were selected for their very quiet operation. This is important because we want to use the digital classroom for teaching and thus need to ensure that the computers are all-but-inaudible even when they are all operating at significant load simultaneously. The computers furthermore consist of an Asus WS X299 Sage motherboard with an Intel Core i7-9800X processor, 32 GB of RAM, a 480 GB Intel Optane 905P SSD, a Sunix parallel port card for interfacing with external hardware using TTL signals and, importantly, a Marvell FastLinQ QL41112HLRJ dual port 10 Gb network card providing high-bandwidth network connectivity. It is important that the computers for each station are identical since this makes it simple to make a disk image of one computer station and then deploy it to another. An unforeseen drawback of our hardware selection should be noted: the heat output of these computer stations is more than the ventilation system of our room can handle. This means that when all computers are running under a load, it may get uncomfortably warm in the digital classroom.

On the desk (Fig. 2), each station is equipped with a Tobii Pro Spectrum 1200 Hz eye-tracker (Nyström et al., 2021), which comes with a built-in Eizo FlexScan EV2451 24″ screen. Each station is furthermore equipped with a RØDE NTH-100M headphone with microphone, a Wooting Two HE keyboard that provides a graded measure of how far keys are depressed, a Logitech G PRO wired mouse which has an ambidextrous design, and a Logitech C922 Pro webcam. Lastly, a Tobii Pro chin- and forehead rest is available for each station. Both the keyboard and mouse were chosen for their low response latency.

The master computer station does not have an eye-tracker since it is meant as a control station for complex multi-station experiments. For instance, this station can be used for controlling simultaneous trial onset across multiple recording stations, or to provide real-time processing and visualization of experiment data recorded on the experiment stations. As such, the computer is largely the same as the other station systems, except that it is equipped with heavier computational capabilities in the form of an Intel Core i9 9920X processor, 64 GB of RAM, and two Nvidia Titan RTX graphics cards equipped with an NVLink bridge.

### Supporting hardware and network infrastructure

**Fig. 3:**
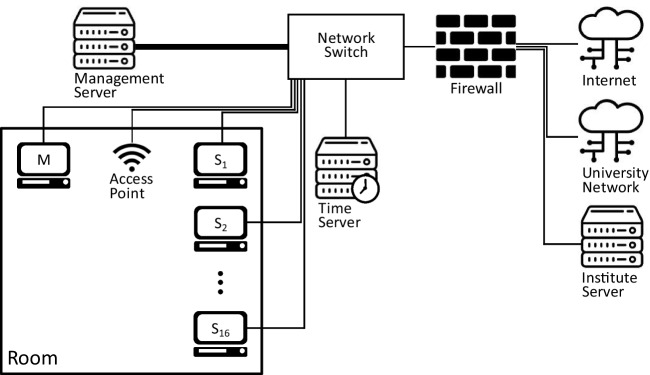
Schematic overview of the computer and network infrastructure of the digital classroom. Inside the digital classroom itself, users have access to a master computer (M), 16 computer stations (S_1_‒S_16_), and a Wi-Fi Access Point. These are connected to other appliances supporting the infrastructure (a management server storing the disk images amongst other things and a time server) with a network switch. The whole infrastructure is connected to the institute’s file servers, the university’s network, and (exceptionally) the internet through a firewall appliance

To enable the hardware in the room to serve as a single setup and meet the goal of providing project environments that are completely under the user’s control, a supporting hardware and network infrastructure has been deployed (Fig. 3 for an overview).

#### Network infrastructure

At the center of this infrastructure is an Aruba 8320 core switch with 48 10 Gb ports and 6 40 Gb ports. Each computer station is directly connected to this switch via the Marvell network card with two uplinks, providing a total bandwidth of 20 Gb/s. To support easy management of the infrastructure by making it possible to remotely start all computer stations without having to walk around and press power buttons, Wake-on-LAN (WoL) functionality should be supported by the network architecture. However, since the core switch only supports ethernet speeds down to 1 Gb/s instead of the 10 Mb/s standby link speed used for WoL, and because the Marvell network cards in the computer stations do not support WoL, a separate management network has been deployed and connected to one of the two network ports onboard each of the computer station’s motherboards. For this management network a D-Link DGS-1100-08P is used as distribution switch and 4× D-Link DGS-1100-05P as leaf switches. Furthermore, to enable wireless clients such as, for instance, eye-tracking glasses to connect to the same network and provide sufficient bandwidth for multiple real-time video streams, an Arista AP-C360 Wi-Fi 6E access point is placed in the digital classroom and connected at 2× 10 Gb/s to the core switch.

#### Security and time appliances

As detailed in the IT security section above, firewalls and virus scanners need to be disabled on the computer stations for reliable performance. To maintain IT security and provide high-speed access from the digital classroom to the rest of the university’s IT infrastructure, a Netgate XG-1541 firewall appliance is used. This firewall is configured such that the university’s license servers, user authentication infrastructure, and the Humanities Lab’s server for storing project data can be reached. The firewall by default blocks access to other resources, such as the internet. Special rules for a specific project can be configured in the firewall to allow access to other resources if a project requires it. Furthermore, the firewall blocks incoming traffic altogether to further reduce the risk of unwanted access to the facility. To provide a reliable, high-quality time source for clock synchronization of the computer stations using the PTP protocol, an Orolia SecureSync 2400 appliance is used.

#### Disk image and management server

Finally, a critical part of the infrastructure is a server for storing the project disk images that can provide these images over the network at a high rate to all stations simultaneously. This server is built around a Supermicro X11SPi-TF motherboard equipped with an Intel Xeon Gold 6132 processor and 192 GB RAM. To ensure sufficient uplink bandwidth to the network, it is connected using both ports of a Marvell FastLinQ QL45412 dual port 40 Gb network card and Aruba 40 Gb QSFP+ LC BiDi MMF transceivers providing a total of 80 Gb/s of bandwidth. To store the project disk images, 4× 12 TB hard drives in a Windows Server storage pool (1 disk redundancy) are used. To ensure that the system is able to saturate its uplink, 2× 3.2 TB Samsung PM1725a SSDs are included in this storage pool (1 disk redundancy) as fast tier, and the significant amount of RAM in the server further serves as disk cache.

### Software

With the above hardware and infrastructure in place, several software tools are needed to bring the facility to life and to deliver separate project environments in a way that involves minimal overhead for staff. The digital classroom makes use of an infrastructure provided by the university and the Lund University Humanities Lab to provide access management and safe storage of project data (such as experiment code and recorded data). The setup furthermore consists of two pieces of software running locally in the digital classroom: (1) Theopenem (theopenem.com), an open-source computer station management system, and (2) labManager (github.com/dcnieho/labManager), a tool consisting of multiple applications that we have developed to perform centralized facility management. Here we briefly discuss how access management is performed, project data is stored, and how we use the Theopenem system, after which we introduce the labManager tool in more detail.

#### Using LDAP for access management

Like many other organizations, Lund University uses an Active Directory infrastructure backed by an LDAP directory service for access management. Information about all users known to the university is stored by the LDAP server, along with what user groups they belong to and associated access permissions. When a researcher or group of researchers want to make use of any of the facilities of the Lund University Humanities Lab, such as the digital classroom, they first apply to start a project in the Humanities Lab. Upon approval, a new user group representing the project is added to the university’s LDAP structure and project members become members of this project group. Both Theopenem and the labManager tools use the university’s LDAP structure for user authentication and to query which projects a user belongs to (if any). By making use of the university’s infrastructure, no credentials or other user data is stored locally. Users can use their regular credentials to make use of the facility—they do not need to remember a new set of credentials, and staff do not have to take any extra action for a user to be able to access the facility.

#### Project data storage

When researchers start a project in the Lund University Humanities Lab, they are provided with storage space for their project. This storage space is intended to be the primary route for users to bring data (such as code to run an experiment) into the lab, and enables data to be accessed from outside the lab’s facilities. As such, in contrast to the digital classroom which is its own firewalled bubble for IT security reasons, this project storage space is accessible from anywhere inside the university. Furthermore, the project storage is an integral part of meeting (inter-)national ethical standards and privacy regulations for data storage, since users have a persistent place to store data that can only be accessed by members of the project. If data were left on the experiment stations, it would be accessible to others using the same facility, or be lost as soon as another project environment is deployed on the computer stations.

#### Theopenem

Theopenem (The Open Endpoint Manager, theopenem.com) is an open-source computer asset management system that provides a system for making and deploying disk images to computers, among other functions. As argued above, for behavioral science lab facilities, the ability to work with such bare metal images is critical. Theopenem provides this functionality by means of a PXE (Preboot eXecution Environment) network boot environment that each of the stations is configured to boot into. If a computer station is configured to be imaged or have an imaged deployed to it, the PXE environment takes care of loading the imaging environment. Otherwise, the computer boots as usual. Theopenem provides access management such that users can only access images belonging to their project, as well as generic clean-slate images provided by lab staff. Importantly, besides a web interface, Theopenem also has a complete API that the labManager software can use to manage a project’s disk images, amongst other things.

#### The labManager tool

Above we have argued that users of multi-user behavioral science lab facilities need a way to create their own project environments that can only be accessed by them. To make such a setup practically workable, users need to be able to easily manage these environments themselves. This becomes even more important once the setup involves multiple stations, such as the digital classroom, since the amount of work multiplies with the number of stations. Using multi-station setups can furthermore be made much easier with tooling for remotely collecting information about the stations, for remotely executing software and other commands on the stations, and for collecting recorded data from all stations and store it in a central location.

**Fig. 4:**
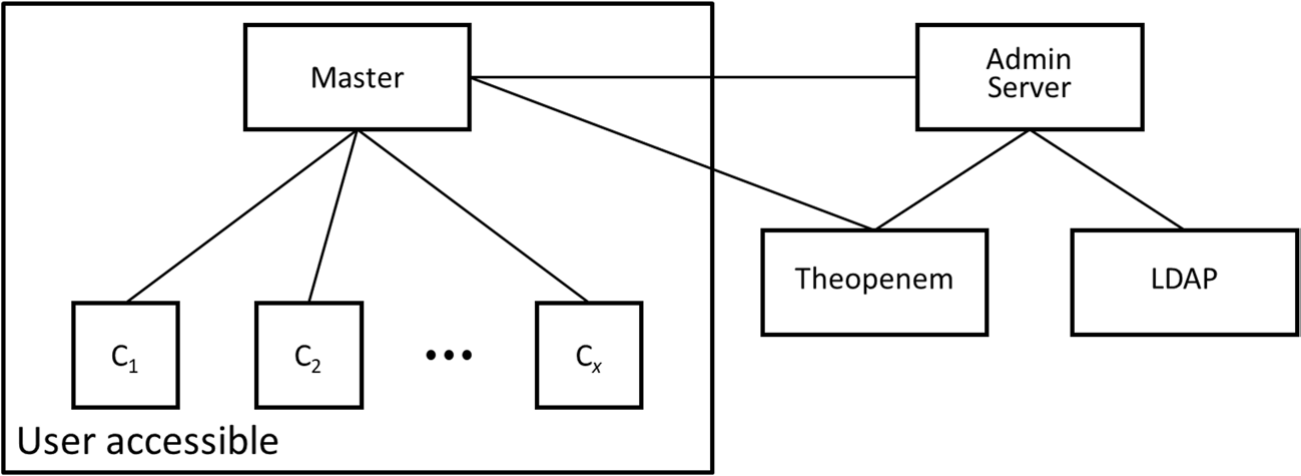
Schematic overview of the labManager tool components and other services they communicate with. In a labManager deployment, users have access to the master application (possibly installed on their own computer) and the client applications (C_1_‒C_16_) running on the stations in the facility. Disk images are stored by Theopenem and user authentication through the institution’s LDAP directory. Since some of the operations on these external services cannot be performed by the user’s account but require elevated privileges, these are handled by the admin server component configured with the required credentials which should be placed on a system that is not accessible to the facility’s users

To provide users with such central management capabilities, we have developed the labManager tool. labManager consist of three parts: (1) a master application from which a user can monitor and control all the computer stations and which disk images are deployed on each, make new disk images, and gather the collected data from all the stations; (2) a client application that runs on each computer station and maintains a connection to the master application from which it receives commands to execute; and (3) an admin server application that provides user authentication capabilities and performs tasks that require elevated privileges that should not be exposed to users. Figure 4 provides a schematic overview of these three tools. The master and client applications support Windows, MacOS, and Linux; the admin server tool has only been tested on Windows.

Inside the digital classroom itself, users have access to a master computer (M), 16 computer stations (S_1_‒S_16_), and a Wi-Fi Access Point. These are connected to other appliances supporting the infrastructure (a management server storing the disk images and a time server) with a network switch. The whole infrastructure is connected to the institute’s file servers, the university’s network, and (exceptionally) the internet through a firewall appliance.

##### labManager master

The core application that users will interact with is the labManager master application. This is an application that can be controlled through a graphical user interface (GUI), or its functionality can be accessed directly as a scripting library by more advanced users. Example scripts for both use cases are provided. labManager master provides users with various capabilities for managing their research environment, the computer stations to which it is deployed, and the data they collect. Here we will describe its functionality, through the lens of a GUI user.

**Fig. 5:**
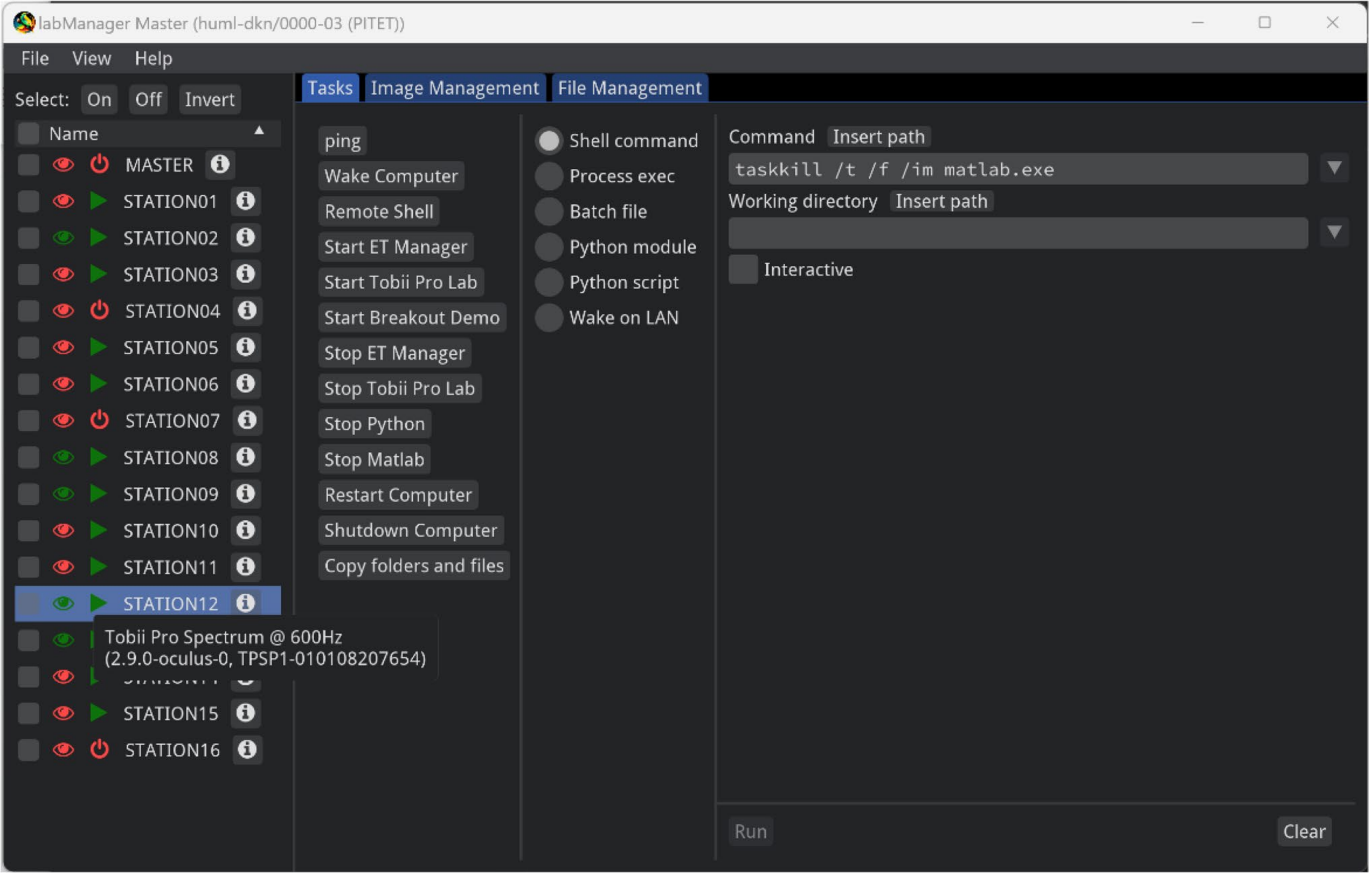
Main interface of the labManager master GUI, when logged into a project. Shown on the left is a list of known computer stations, along with a visual representation indicating which stations are connected to the master application (green triangle) and which stations have a connected eye-tracker (green eye). Information about one of the connected eye-trackers is shown. On the right side of the GUI, the Tasks pane is activated, which consists of three sections. On the left is a set of pre-configured tasks; pressing one of the buttons prefills the middle and the right panels. The middle panel is the type of task, such as executing something on the remote station(s) command line, or running a Python script. The right panel is used to configure the details of the task, such as the command to execute

When starting the application, users are asked to log in using the same credentials as they use for other university services and are then presented with a list of projects in which they are a member. After selecting the project they want to work with, the main interface opens (Fig. 5). On the left, this interface shows a list of known computer stations, along with information about whether these stations are running, and information about the eye-tracker connected to each of the stations. Unknown stations, if any, that connect to the master are also added to this list. On the right, a user can choose to show one of four views.

The first view (shown in Fig. 5) is an interface for launching tasks on the stations. This is done by selecting one or multiple target stations on the left and either selecting a preconfigured task from the list or configuring your own task. This task is then executed by pressing the run button. Tasks include a WoL action (the only task that can be executed on stations that are currently not running), the running of a shell command, the running of an executable, and the running of a Python script or module. Additional arguments to each of these tasks can be provided, and the working directory in which they should be executed can be configured. Finally, a task can be made interactive, meaning that once started, the user can provide additional input to the task. For example, this makes it possible to have a remote shell (command line) session running on the station that receives input from the master computer.

**Fig. 6:**
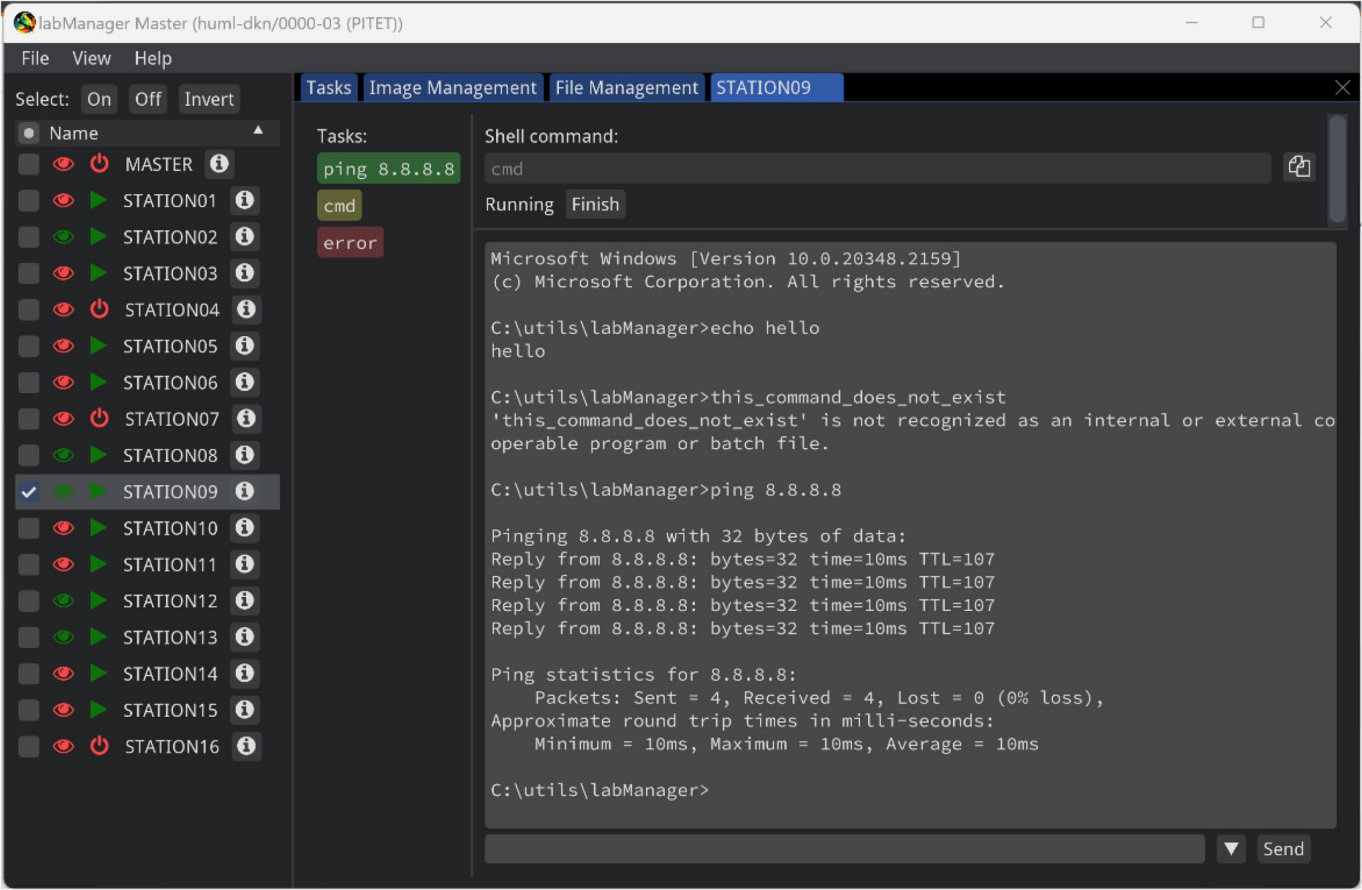
Station view. In this view, the status or result of executed tasks is shown. In the example, the output generated during a remote shell session is shown

The second interface is a station view (Fig. 6). In this view, eye-tracker events (e.g., changes in its configuration) and previous or currently running tasks are shown. By clicking on these events or tasks, more information about the task is shown and the task can be stopped or (in case of an interactive task) interacted with if it is still running.

**Fig. 7:**
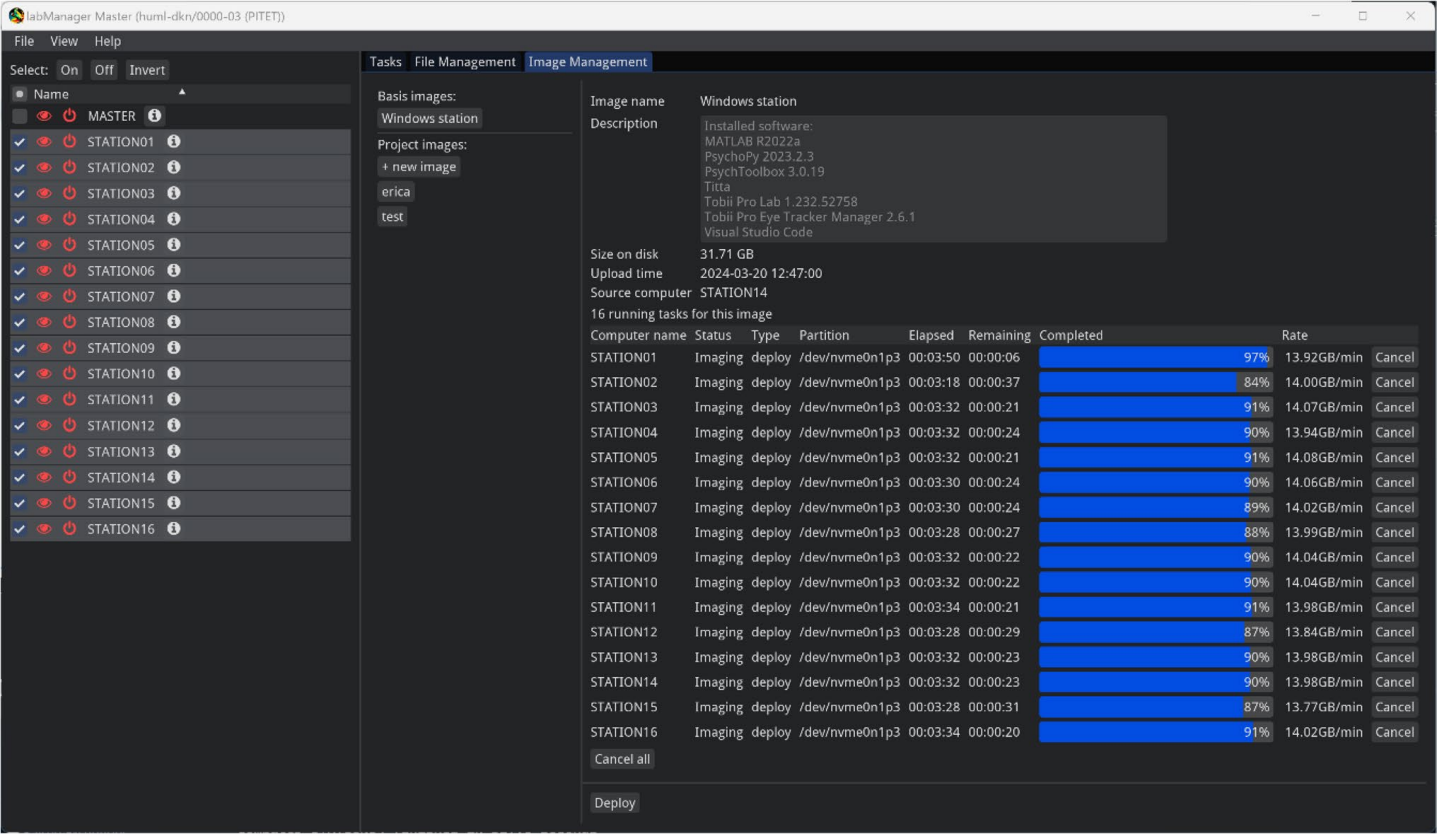
Imaging view. Shown in the middle panel is a list of basis disk images provided by facility staff, along with an interface for managing images specific to the logged-in project. The right panel provides details about a selected image, and makes it possible to deploy, upload, or delete it. Shown is a deploy action for an image to multiple stations

The third interface is the imaging view (Fig. 7). In this view, users can manage the disk images that belong to their project, as well as deploy base images provided by staff. A typical workflow for creating and managing a project’s disk images is discussed below under Workflow. In addition to being able to create and deploy disk images, as shown in the figure, this view also shows the progress of any running imaging tasks and makes it possible to cancel them.

**Fig. 8:**
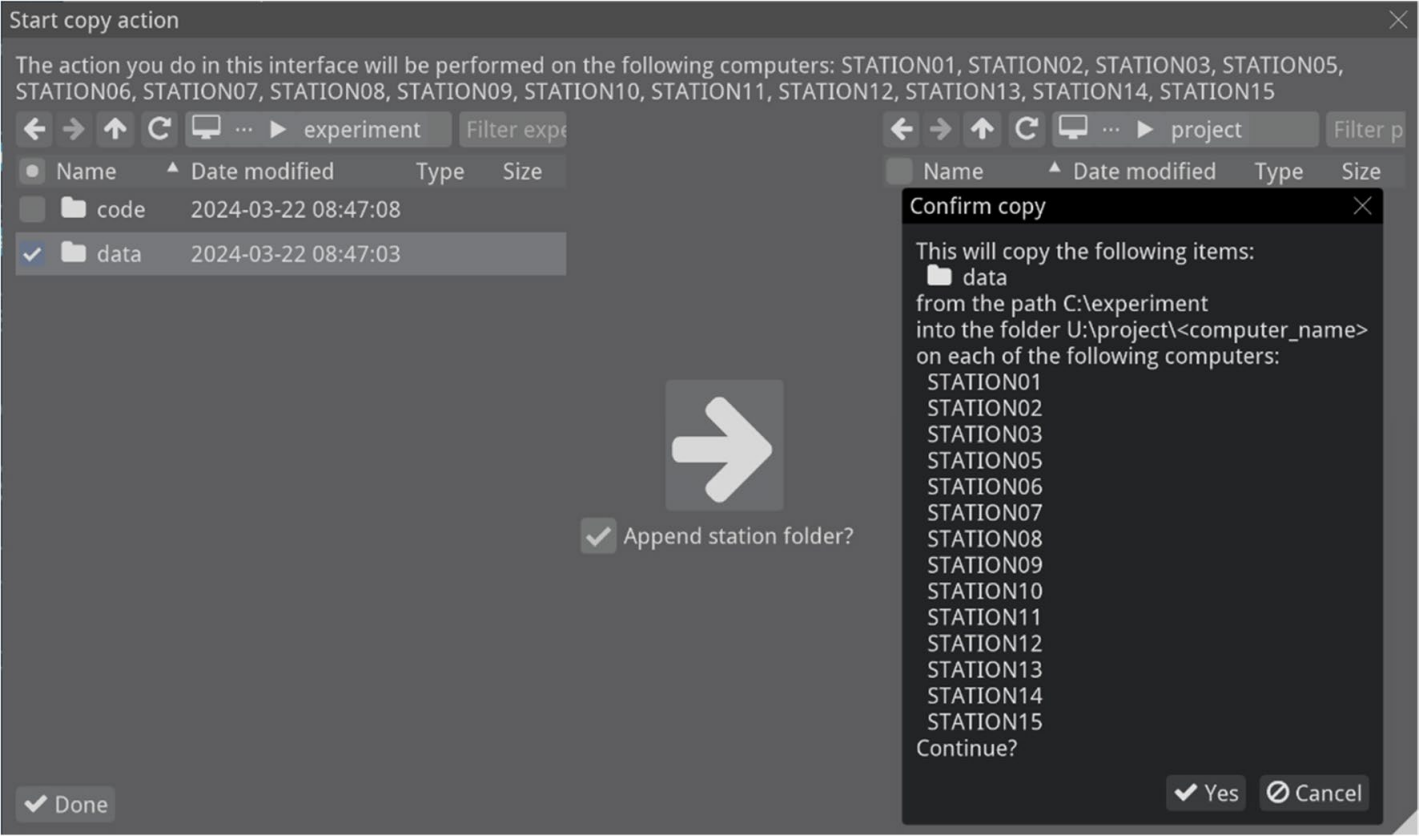
File management view. Shown is a confirmation popup for a copy action from a source directory on the left to a destination directory on the right, set up such that the data from each of multiple selected stations is put in its own subfolder with the station name

The last interface is the file management view (Fig. 8). In this view, users can launch file and folder operations on the stations. For instance, this enables users to create a folder on all the stations, copy experiment code from a central location to that folder on all the stations, and once data is collected, copy the data from all the stations to a central location. The interface also makes it possible to automatically make a folder with each station’s name and per station copy files into that station’s folder. That way, when files are copied to a central location, they are separated by stations, and files with the same naming do not lead to clashes and potentially lost data.

##### labManager client

The labManager client is an application that runs on each station in the facility and that the user will rarely interact with. It should be configured to automatically start when a station boots. Once started, the client will periodically query the presence of any labManager master applications. The client will connect to any discovered masters. Once connected, the client provides information about the station to the master and will execute any commands received from the master. It also listens for any connected Tobii eye-trackers and monitors changes in their configuration to report back to the master.

##### labManager admin server

The final part of the labManager tool is the admin server. The admin server handles tasks that require elevated privileges. Currently this tool is designed to handle the verification of user credentials against the institute’s LDAP server, and to perform disk image management tasks in Theopenem that require elevated privileges (fine-grained logic to verify that a user is allowed to execute the requested action is implemented in the admin server). To do its job, the admin server needs to be configured with the credentials for special accounts, and as such the admin server is meant to be run on a system that is not accessible to users.

### Workflow

Here we will briefly discuss the workflow for using the labManager tools, both from the perspective of lab facility staff, and for facility users.

#### Facility staff

Most of the work for facility staff is in the initial set up of the labManager tools. This consists of deploying Theopenem and the labManager admin server to a suitable secure system, and providing it with the required credentials to the Theopenem installation and the institute’s LDAP instance. Furthermore, for ease of use it may be practical to provide a computer (possibly a tablet) in the facility that is set up with the labManager master, so that users do not have to set it up themselves when they wish to make use of the facility. The labManager master should also be configured such that it knows how to connect to the labManager admin server, and optionally to contain a list of known computer stations and pre-filled actions to perform on them. Example configuration files are provided with the labManager tools.

Finally, although not required, it is recommended that facility staff provide a disk image that users of the facility can use as a basis for their own project environment. This image should have the labManager client application integrated, and as a service may include other tools commonly used in the facility. The only regular work for facility staff is to update the software and operating system provided in this disk image, so that new projects start with up-to-date software, a fully patched operating system, and the latest drivers. Importantly, such updates do *not* affect existing project environments already created by users, since these are stored in their own disk images.

#### Facility users

If the facility staff provide a basis disk image that contains the software that many of the facility’s users need (see Facility staff), the first step that a researcher starting a new project would likely perform is to log into labManager master and deploy this base image to one of the computer stations. The researcher can then customize this image to their needs, for instance by installing software they need, changing settings, and deploying the code for their experiment or recording. In the tool, they can then create a new disk image within their project, and start an upload of the station they have prepared to this disk image. Once this disk image has been completed, they will be able to deploy it to any of the stations in the facility. If the user later makes changes to their setup, they can then overwrite this disk image, or store it as a new image in their project, if they prefer.

If the researcher wants to perform data collection, after logging in to the labManager master, they deploy their disk image to the station(s) they want to use. To make it easy for the researcher to ensure that they have the right environment deployed to the stations and the right peripheral hardware setup, labManager master shows what image is deployed on each of the connected stations, and provides information about the firmware version on a connected eye-tracker (if any) as well as some of its settings. The researcher can remotely issue a command to the computer stations to run their experiment, and once finished, they can use the labManager master’s file manager to collect all their recorded data and copy it to a single location, such as the central project data storage server that we provide for our user projects. To give users easy access to this storage location, it is automatically mounted as a network drive on each of the stations when the labManager client on the station connects to the master.

### Policy

Experience has shown us that certain policies are required for the smooth functioning of lab facilities. All activities in the lab are organized as projects. All projects require a user agreement that is signed both by the researcher responsible for the project and by lab management. This ensures that users are aware of our policies, and of the responsibilities of staff and users, respectively. A discussion of the user agreement form enables us to clarify these responsibilities to the users, to ascertain that the facility can indeed provide the hardware and software that the project requires. It provides staff with an inventory of each project’s needs and any special accommodations that are required and have been agreed upon.

From the perspective of avoiding wasting resources, it is also important to ensure that users have the necessary expertise to carry out their project, and some labs also at times vet project plans to ensure their scientific merit. Before the project agreement meeting, a prospective user should therefore also consult with the relevant facility and method experts to discuss their project idea and needs. This discussion provides lab staff with an overview of what projects are active and allows for the planning for maximum accessibility to the facility when multiple projects are running in parallel.

While some of the responsibilities and obligations for staff and users are specific to our local circumstances and will thus not be discussed, we think it worthwhile to highlight a few. From the lab’s side, the facility commits to providing the user with the necessary hardware, software, and computing power for their project, as well as centralized storage space (see Project data storage) for the duration of the project. Users are responsible for immediately moving recorded data to this project storage space since that is the only storage provided for the duration of the project. Data is removed from the lab servers upon completion of the project, and the researcher is then solely responsible for handling their data in compliance with any long-term storage guidelines that apply. It is also made clear to the researcher that they alone are responsible for the research design, verification of the hardware setup, data collection, analyses, and compliance with good ethical practice. Users are therefore responsible for ensuring that they have performed sufficient piloting of both their experiment and their analysis to ensure that all the data they need is indeed recorded. Lab staff are not obliged to assist in any of these tasks, or to collaborate with the researcher, beyond ensuring that equipment is in good working order.

## Evaluation

An important design goal for our facility and software tools is that it should be possible to quickly create and deploy disk images of users’ project environments, such that this stage does not become a significant impediment to using the facility. A further design goal was to allow for the sending of data between different stations with low latency. In this section we present two evaluations that center on these capabilities. Both evaluations were performed with the timing_instrumentation branch of labManager (v1.0.5), available from https://github.com/dcnieho/labManager/tree/timing_instrumentation. The exact version used is archived at 10.5281/zenodo.11582817.

### Creating and deploying disk images

The creation of disk images, and especially the deploying of these disk images to all the computer stations, should be fast so that it does not cause significant waiting time each time a user wants to run their project in the facility. In particular, deploying the disk images should not take long since it must be done every time a user enters the facility, for instance before the start of each data collection session. Somewhat arbitrarily, we have therefore aimed for the deploying of the disk images to take about 5–10 minutes. The creation of disk images would presumably occur less frequently than the deploying of disk images, but nevertheless would ideally take about the same time.

To examine how long these two actions take, we have therefore run two tests. The first test comprised the simultaneous creation of two disk images from two different stations, such as may occur in a setup consisting of a control station and one or multiple experiment stations. Each station contained a Microsoft Windows Server 2022 installation that took up 55.4 GB of disk space (Windows itself along with device drivers and several installed programs such as Tobii Pro Studio, PsychoPy, and MATLAB). Image creation time was measured from the moment the command to create the disk images was given from the labManager master interface until the disk image creation was completed. For the two stations this took 7:50 and 7:54 respectively, while during the imaging approximately 10.8 GB/min was transmitted to the disk image server. Since the two computer stations to be imaged were switched off when the command was issued and started through a Wake on LAN message, the measured time includes the time it took to boot the stations.

The second test comprised the deploying of a disk image to all 16 stations in the digital classroom simultaneously. Again, all systems were switched off when the imaging action was launched, and the measured times thus include the time it took for the systems to boot into the imaging environment. Imaging the stations took between 4:50 and 5:19, and imaging speeds of around 14 GB/min were observed for all stations.

In summary, both the creation of disk images and the deploying of these disk images to the computer stations took no longer than it takes to grab a cup of coffee, and should therefore not constitute a significant hold-up for most uses of the experiment facility.

### Communication latency

We also examined how much time it took to send data between stations (i.e., the communication latency) and the variance of this latency. Knowing the latency and how this latency depends on the size of the messages helps decide which applications requiring inter-station communication are possible to conduct with the setup. While some experiments, like joint search tasks (Niehorster et al., 2019), require low latency and low variability (constant data rates), for other experiments it may be unproblematic if data arrives with a variable and significant latency.

To test the latency with which messages can be sent and received between stations, we developed two Python scripts, a server script which can send data of variable size at variable rates to multiple clients, and a client script which can receive such data from multiple clients. We opted to use TCP connections, since such connections are reliable, which means that they do not experience data loss. Since operating system-level multithreading is not possible in a single Python script, which limited our ability to handle many clients from one script, multiple server and client scripts were started on each station, and each handled a subset of the connections. Specifically, each station both sent data to the other 15 stations and simultaneously received data from all of them. We configured the script to, for 1 minute, send 16-, 64-, 256-, 1024-, or 5120-byte messages at 1200 Hz. For context, a message of 336 bytes would correspond to the maximum number of data streams that a Tobii Pro Spectrum eye-tracker could produce, and 1200 Hz is its maximum sampling frequency. The bigger message sizes were used to further test the network under higher load conditions. To test it even further, two more 1-minute tests were performed using 1024- or 5120-byte messages at 4800 Hz. This last condition created about 3.0 Gbps inward and 3.0 Gbps outward traffic that was handled simultaneously. This is well in excess of what even multiple webcam streams would generate (for context, a 4K highest-quality Netflix stream consumes about 15 Mbps). The scripts were automatically run and the data automatically copied to a central location using the scripting ability of the labManager tools. All code for this evaluation is available at https://github.com/dcnieho/labManager/tree/master/paper_tests/communication_latency.

Analysis of this data was performed using the method of Nyström et al., 2016, (see their Fig. 5 and related text) to assess communication latency regardless of offsets between the clocks of the two systems. Briefly, the analysis worked as follows. High-resolution timestamps were acquired using the *win-precise-time* library (v1.4.2, measured to provide a granularity of 238 ns on our systems) both just before a packet was sent from one station, and directly after it was received on the other station. For each measurement session and each pair of stations, the difference in receive and sent timestamps (Δ*t*) for each message was calculated for all messages in both directions (*a*->*b* and *b*->*a*). Following the analysis method of Nyström et al. (2016), straight lines were fit to these two timeseries using the MATLAB *robustfit* function, using the default Huber distance norm. The end-to-end communication latency between the two stations was then given as the mean of the intercepts of the two fits. To determine variability in the latency, the mean absolute deviation (MAD) of the latency data around the fit line was furthermore calculated.

**Fig. 9:**
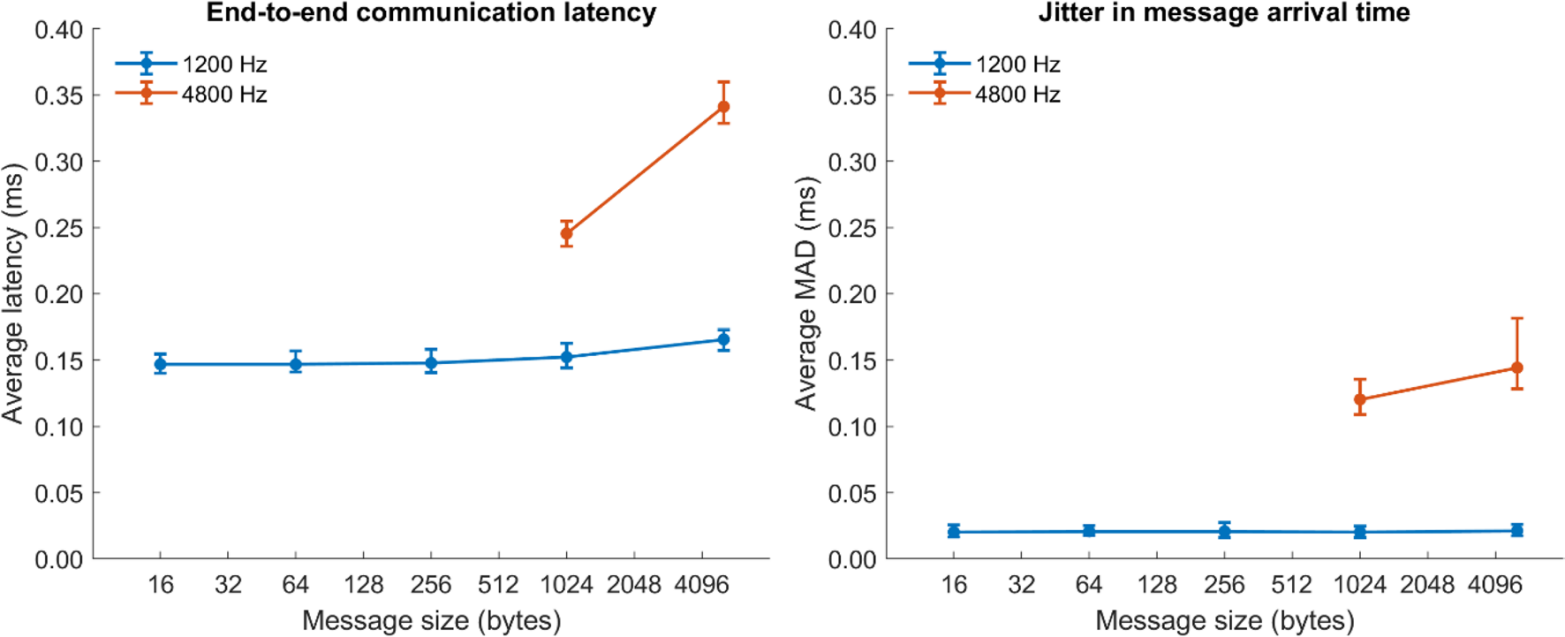
Average end-to-end communication latencies (left panel) and jitter in message arrival times (right panel) as a function of message size and data rate. Error bars represent 95% confidence intervals

Average end-to-end communication latency (including network and processing latencies) and jitter in message arrival time are shown in Fig. 9. As can be seen, even at the highest data rates, the communication latency and the jitter in message arrival time is minimal (both well under half a millisecond). We presume that this is sufficient performance for most applications.

## Discussion

In this article, we have sketched a problem that many users and staff of multi-user lab facilities focusing on a behavioral science context face, namely, the safeguarding of experiment integrity and data safety in the face of multiple users with conflicting needs. We have provided an extensive discussion of possible solutions to this problem, along with preconditions that these solutions should meet. We have furthermore presented a hardware and software (The labManager tool) solution for this problem deployed at the Digital Classroom in the Lund University Humanities Lab, along with tests. Importantly, our solution provides each user with a completely separate computer environment that is easily preserved and restored and which they are free to set up according to their needs.

Does our solution mean that we can now declare the problem of running and using multi-user lab facilities fixed? No, several outstanding issues remain. The first issue is hardware, where both firmware versions and on-device configuration are outside the realm of our solution. This not only concerns the firmware deployed to a connected eye-tracker or other specialist device, but also includes settings stored on peripheral devices such as mouse sensitivity and keyboard latency mode that may be critical for the proper functioning of some setups.

Second, while our solution provides users with all the tools, the proper storage of data remains the responsibility of the user even in our setup. A user who does not copy the data from the computer stations to the central secure storage facility risks losing their data, and a user who does not delete their data from the computer stations after copying it leaves it exposed to others with access to the facility. While the latter (but not the former!) risk could be mitigated by requiring a lab user to log in to each computer station, this would make using the facility unwieldy (or even impossible if a researcher brings along a research assistant but does not want to disclose their password) and does not protect users from nefarious intruders who could, for instance, extract the hard drives containing the data from the computer stations.

### Single-station setups

In this paper we have described the setup of the Lund University Humanities Lab’s Digital Classroom. This setup is complex, and several components (such as the network equipment and management server) are costly due to the performance required for supporting a setup consisting of many computer stations. Is our solution suitable for simple, far more common, setups consisting of only a single multi-user experiment station? Yes, we believe it is. Nothing in the design of the labManager tools requires the presence of more than one computer station in a setup. Such a single-station setup would furthermore be well served with much simpler management hardware than described under Supporting hardware and network infrastructure. A simple, relatively cheap server computer along with a gigabit switch should be sufficient to run such an environment, and would require no special hardware in the experiment station. It should be noted that deploying a disk image would take about twice as long as reported above due to the speed limitation of a gigabit network interface. However, this bottleneck could be resolved using a 2.5-gigabit network setup. The increase in cost over a gigabit setup is likely low, since 2.5-gigabit network ports are becoming more common on consumer-level computers, and network switches supporting such speeds can be bought for under 100 USD.

There are also many facilities where there are multiple separate experiment setups, for instance placed in multiple rooms. The labManager tools can also be used without modification for managing such facilities. Depending on the number of setups, it may be necessary to invest in a server computer that is able to stream disk images at multi-gigabit speeds, and in a network switch with uplink ports that are able to handle these multi-gigabit speeds. As discussed above, single-gigabit or 2.5-gigabit connections to the experiment stations in the individual rooms should be sufficient. Furthermore, it is a good idea to configure the network switch such that network traffic between the setups is not possible. This will make it impossible for a user of one setup to deploy a disk image on the experiment station of another setup, and such separation is also desirable for IT security. With suitable network configuration, a single management server and labManager installation can serve these different separated experiment setups.

### Multiple participant use

Is our hardware setup and the labManager toolset all that is needed to run and use a multi-user behavioral science lab? That depends on the use case. Returning to our example users introduced above, most would be well served by the currently provided set of tools, while others would need further specialist tools. Their use cases can be broadly divided in two:

1. Researchers who use the facility as a set of separate stations for quick parallel data collection, to run classroom-size experiments that do not require interaction between stations, or for teaching a hands-on eye-tracking course (the visual arts researcher, the communication scientist, and the teachers; users 2, 4, and 5).
2. Researchers who have the additional requirement that they want to use some form of technology-mediated interaction for their study, and thus need to stream and visualize data across computer stations (the computer and cognitive scientists; users 1 and 3).

For the first class of use cases, the tools and physical setup discussed serve users all the way. It enables them to craft their setup on one computer station, which can then easily be replicated to other stations and be used to achieve massive data collection speed-up though parallelization. However, the second class of cases, with users who want to tap into the unique opportunities that multi-station facilities offer, are only partially served by the labManager tools. While such users are able to use the labManager tools to ensure that the correct setup is deployed to the correct computer stations (such as, e.g., stations of type A, sender of data, stations of type B, receiver of data, and a master station directing the whole experiment), labManager does not provide a means for them to achieve inter-station communication. Such needs will need to be subserved by other tools, such as LabStreamingLayer (labstreaminglayer.org) and a recent extension to the Titta toolbox (Niehorster et al., 2020) to stream data of Tobii eye-trackers using LabStreamingLayer (github.com/dcnieho/Titta/tree/master/LSL_streamer).

Thus, while there remains work to be done for some use cases, we believe that the blueprint we provide for an infrastructure design, along with the open-source labManager tools introduced here are important steps towards ensuring maximum flexibility for users, data security, and ethical compliance, while simultaneously offering ease of management. This work thus paves the way for important improvements to multi-user facilities in the behavioral sciences.

## Data Availability

Not applicable.

## Footnotes

1 https://www.humlab.lu.se/education/commissioned-education/

## Code availability

The labManager tools are available at https://github.com/dcnieho/labManager.
